# Nitrate of Silver in Tonsilitis

**Published:** 1835

**Authors:** 


### VI. — Nitrate of Silver in Tonsilitis.

It is well known that no disease is more likely to recur than suppurating tonsilitis, which, under the name of quincy, will return upon some persons, on the slightest exposure to a cold and damp atmosphere, subjecting him to repeated suppurations. It is perhaps not generally known, that on the access of this malady a vigorous and early application of lunar caustic, in the solid form, or dissolved in rain water, in the proportion of eight or ten grains to the ounce, will more certainly arrest the inflammation and prevent an abscess, than any other method of treatment. If, however, there should be fever, venesection and an emetic should be premised. The application of solid caustic to the tonsils until they "turn pale," gives but little pain. If it should escape from the quill and be swallowed, it may be decomposed with salt and water, or a solution of sulphate of magnesia.

